# Neoadjuvant Chemotherapy Following Hyperthermic Intraperitoneal Chemotherapy in Platinum-Sensitive Recurrent Ovarian Cancer: A Retrospective Cohort Study

**DOI:** 10.3390/cancers18050744

**Published:** 2026-02-26

**Authors:** Alberto Rafael Guijarro-Campillo, Alida González-Gil, Elena Gil-Gómez, Vicente Olivares-Ripoll, Javier Sánchez-Romero, Jerónimo Martínez, Aníbal Nieto, Pedro Antonio Cascales-Campos

**Affiliations:** 1 Peritoneal Carcinomatosis and Sarcomas Unit, Hospital Universitario Virgen de la Arrixaca, IMIB-Arrixaca, 30120 Murcia, Spain; 2 Department of Gynecologic Oncology, Hospital Universitario Virgen de la Arrixaca, IMIB-Arrixaca, 30120 Murcia, Spain; 3 Department of Surgery, Hospital Universitario Virgen de la Arrixaca, IMIB-Arrixaca, 30120 Murcia, Spain; 4 Department of Medical Oncology, Hospital Universitario Virgen de la Arrixaca, IMIB-Arrixaca, 30120 Murcia, Spain

**Keywords:** neoadjuvant chemotherapy, secondary cytoreductive surgery, hyperthermic intraperitoneal chemotherapy

## Abstract

Objective: This study aims to compare the efficacy of neoadjuvant chemotherapy (NACT) followed by secondary cytoreductive surgery (SCS) combined with hyperthermic intraperitoneal chemotherapy (HIPEC) versus SCS with HIPEC alone in patients with platinum-sensitive recurrent ovarian cancer. Methods: A retrospective analysis was conducted on patients treated at Hospital Clínico Universitario Virgen de la Arrixaca from January 2011 to December 2024. Two cohorts were analyzed: those receiving NACT prior to SCS and HIPEC, and those undergoing SCS with HIPEC without prior NACT. The primary endpoint was progression-free survival (PFS), while secondary endpoints included overall survival (OS), peritoneal progression-free survival, extraperitoneal progression-free survival, and safety. Results: A total of 86 patients were included in the study, with 39 in the NACT group and 47 in the non-NACT group. The non-NACT cohort exhibited a significantly longer PFS of 20 months compared to 12 months in the NACT group (*p* = 0.004). Median OS was also superior in the non-NACT group, at 88 months versus 34 months (*p* = 0.023). Patients in the NACT group experienced longer surgical durations, increased transfusion requirements, and a higher incidence of severe complications. Conclusions: The addition of NACT prior to SCS and HIPEC does not enhance survival outcomes for patients with platinum-sensitive recurrent ovarian cancer. In fact, those who underwent SCS with HIPEC alone demonstrated better PFS and OS, underscoring the importance of careful patient selection in optimizing treatment strategies.

## Simple Summary

In this study, we examined the role of neoadjuvant chemotherapy (NACT) followed by secondary cytoreductive surgery (SCS) combined with hyperthermic intraperitoneal chemotherapy (HIPEC) in patients with platinum-sensitive recurrent ovarian cancer. Our findings indicate that patients who did not receive NACT exhibited superior progression-free survival (PFS) of 20 months compared to 12 months in the NACT group. Additionally, those receiving NACT faced longer surgical durations and a higher incidence of complications. These results emphasize the critical need for careful patient selection in treatment strategies to enhance outcomes in this patient population.

## 1. Introduction

Ovarian cancer remains a leading cause of mortality among gynecologic malignancies, with a significant number of patients experiencing recurrence despite advancements in treatment modalities [1,2,3,4,5]. Approximately 70% of women diagnosed with advanced-stage ovarian cancer will relapse within three years of initial management, presenting a substantial clinical challenge [6,7].

The randomized DESKTOP III trial [8] and the partially completed SOC-1 trial [9,10] have demonstrated a survival advantage for secondary cytoreductive surgery (SCS) followed by chemotherapy compared to chemotherapy alone, with the most favorable outcomes observed in patients achieving complete gross resection. Achieving no visible residual disease is optimal when the correct patient selection for SCS is performed; however, the selection criteria established in the DESKTOP III and SOC-1 trials are not universally applied in clinical practice [11].

Recent investigations have examined the role of neoadjuvant chemotherapy (NACT) followed by SCS combined with hyperthermic intraperitoneal chemotherapy (HIPEC) in patients with platinum-sensitive recurrent ovarian cancer. The randomized controlled CHIPOR trial has shown improved overall survival in epithelial ovarian cancer patients with first platinum-sensitive relapse treated with second-line platinum-based chemotherapy followed by complete secondary cytoreduction and HIPEC compared to those not receiving HIPEC (overall survival HR 0.73, 95% CI 0.56 to 0.96, *p* = 0.024) [12]. The median overall survival was 54.3 months in the HIPEC group, compared to 45.8 months in the non-experimental group, aligning with the median overall survival reported in the DESKTOP III trial [8]. This suggests that both treatment strategies are viable options for this patient population; however, the availability of two effective treatments raises questions regarding the optimal choice for patients, particularly within the context of well-consolidated secondary cytoreductive surgery. Conversely, the HORSE trial, which evaluated HIPEC during SCS without prior NACT, failed to demonstrate a significant benefit in progression-free survival (PFS), casting doubt on the necessity of HIPEC in all contexts [13]. While both studies provide valuable insights into managing recurrent ovarian cancer, their inherent design limitations and the absence of direct comparisons highlight the need for further comprehensive investigation.

This study aims to compare two distinct cohorts of patients with recurrent ovarian cancer: one receiving NACT followed by SCS with HIPEC, and the other undergoing SCS with HIPEC without prior NACT. The rationale for this comparison stems from the lack of data regarding the optimal timing of surgery in the recurrent setting, which is crucial for improving outcomes in this patient population.

## 2. Methods

### 2.1. Accrual and Data Source

This is a retrospective observational single-center study aimed at comparing the efficacy of two treatment approaches in patients with platinum-sensitive recurrent ovarian cancer at Hospital Clínico Universitario Virgen de la Arrixaca, Murcia, Spain, from January 2011 to December 2024. Two groups were analyzed: the first group consisted of neoadjuvant NACT followed by SCS and HIPEC, while the second group included SCS and HIPEC without prior NACT. All patients were evaluated within a Multidisciplinary Tumor Board. The REG-COP-2020-01 project, approved by the Institutional Review Board, aims to build and manage a retrospective database of patients from the Peritoneal Surface Malignancies Unit and is centralized in the Spanish Peritoneal Oncological Surgery Registry (GECOP). The study was performed in accordance with the Helsinki Declaration of Ethical Principles. All patients provided written consent. All study researchers complied with data confidentiality in accordance with General Data Protection Regulations. Data are collected anonymously and confidentially, ensuring compliance with current data protection legislation (GDPR and LOPDGDD).

### 2.2. Patients Selection

Patients were included if they had recurrent epithelial ovarian, fallopian tube, or primary peritoneal cancer classified as platinum-sensitive, defined by a platinum-free interval of at least six months following the completion of first-line chemotherapy, with the disease limited to the abdomen and no distant metastases. The site of recurrence was classified by location as peritoneal, extraperitoneal, or mixed. Eligibility for secondary cytoreductive surgery (SCS) was determined based on imaging and clinical evaluations. All patients were required to have a positive Arbeitsgemeinschaft Gynäkologische Onkologie (AGO) score, indicating a favorable prognosis for complete resection, and to be aged between 18 and 80 years. Additionally, they needed to have an Eastern Cooperative Oncology Group (ECOG) performance status of less than 2 and to meet the absence of absolute unresectability criteria as defined in the 2019 European Society of Gynaecological Oncology (ESGO) guidelines [1]. Patients also needed to fulfill the International Federation of Gynecology and Obstetrics (FIGO) criteria, with the FIGO stage assessed at the initial diagnosis. Exclusion criteria included prior anti-angiogenic therapy within eight weeks before surgery and non-epithelial ovarian histology.

### 2.3. Procedures and Follow-Up

The decision to proceed with neoadjuvant chemotherapy (NACT) or secondary cytoreductive surgery (SCS) was based on the feasibility of performing SCS within an optimal timeframe of four weeks. As our center serves as a reference for ovarian cancer treatment across a broad region, many patients referred to us may not meet this timeframe, leading to the initiation of adjuvant treatment instead. Patients in the NACT group received between four to six cycles of platinum-based (carboplatin/cisplatin plus paclitaxel) NACT prior to surgical intervention, in accordance with the inclusion criteria established by the CHIPOR trial. Following a subsequent assessment of their response via imaging, and to ensure appropriate patient selection in the NACT group, certain inclusion criteria that may have been affected by NACT (such as ECOG performance status, AGO score, and ESGO criteria for unresectability) were re-evaluated prior to surgery.

All patients received standardized perioperative management, which included bowel preparation, prophylactic antibiotics, and thromboprophylaxis. Surgical procedures were tailored based on tumor extent, location, and surgeon discretion. A xiphopubic laparotomy was performed on all patients to evaluate the entire peritoneal cavity, and the extent of disease was quantified using the Peritoneal Cancer Index (PCI). Peritonectomy procedures were defined according to the latest subdivisions and boundaries established by consensus among the Peritoneal Surface Oncology Group International (PSOGI), the European Society of Gynaecological Oncology (ESGO), and the International Society for the Study of the Pleura and Peritoneum (ISSPP) [14]. HIPEC was administered with Paclitaxel (60 mg/m^2^ body surface area) from the inception of the peritoneal oncologic surgery program in 2008 until January 2012, when our group transitioned to Cisplatin (75 mg/m^2^ body surface area) as part of a prospective, randomized study designed to evaluate survival outcomes after cytoreductive surgery (CRS) with or without HIPEC in patients with IIIC–IV ovarian cancer who had received NACT [15]. In both cases, intraperitoneal treatment was administered at a temperature of 42 °C for a duration of 60 min. After surgery, patients in the second group received platinum-based adjuvant chemotherapy until a total of six cycles were completed. The completeness of cytoreduction was assessed using the completeness of cytoreduction score (CC), where CC-0 indicates no residual tumor and CC-1 indicates residual tumor measuring less than 0.25 cm. Patients with significant residual disease were excluded from the analysis. Following hospital discharge, all patients were evaluated every three months during the first two years with comprehensive analyses and CT scans, and subsequently every six months.

### 2.4. Outcomes

The primary endpoint was progression-free survival (PFS), defined as the time from recurrence to secondary recurrence, disease progression, or death from any cause, whichever occurred first. Disease recurrence was identified using imaging methods, including computed tomography, magnetic resonance imaging, and positron emission tomography/computed tomography, supported by clinical examination and tumor markers.

Secondary endpoints included overall survival (OS), defined as the time from recurrence to death from any cause. Additional secondary endpoints outlined in the statistical analysis plan encompassed peritoneal progression-free survival, extraperitoneal progression-free survival, the pattern of relapse at the time of secondary recurrence, and safety. Peritoneal progression-free survival was measured as the interval from recurrence to the occurrence of peritoneal progression, indicated by clinical signs of a peritoneal mass or effusion with cytological confirmation, or a detectable peritoneal mass through imaging. This was censored at the time of death or the last follow-up if no peritoneal progression was observed. Similarly, extraperitoneal progression-free survival was defined as the time from recurrence to the detection of extraperitoneal progression, assessed through clinical examination or imaging, also censored at death or the last follow-up in the absence of such progression.

The severity of perioperative complications was assessed using a 1–5 scale based on the Surgical Secondary Events Grading System from the Memorial Sloan Kettering Cancer Center (MSKCC) (New York, United States) [16]. Toxicity was evaluated according to the Common Terminology Criteria for Adverse Events (CTCAE) version 6.0 [17].

### 2.5. Statistics

Differences in categorical variables were analyzed using the χ^2^ test. In cases where expected values were insufficient, Fisher’s exact test was applied. Continuous variables were summarized using medians and interquartile ranges (IQR), with comparisons made using a non-parametric test, specifically the Mann–Whitney U test, due to the unlikely normal distribution of the data. Survival analyses were conducted using the Kaplan–Meier method, with comparisons between groups assessed using the log-rank test. Subgroup analyses were conducted for exploratory purposes to assess the consistency of the treatment effect across clinically relevant variables, including completeness of cytoreduction score (CC-0 vs. CC-1), the interval between the completion of primary therapy and recurrence (6–12 months vs. >12 months), age (<65 years vs. ≥65 years), peritoneal cancer index (PCI < 5 vs. ≥5), histology (high-grade serous vs. other types), FIGO stage (I–II vs. III–IV), number of peritonectomies during SCS (<2 vs. ≥2), and breast cancer gene (BRCA) mutation status (mutated vs. non-mutated). Given the limited sample size, no adjustment for multiple comparisons was performed. Multivariate analyses utilizing the Cox proportional hazards model evaluated the treatment effect of statistically predefined subgroups. All analyses were performed using Stata/BE 18.0 (StataCorp., College Station, TX, USA). Statistical significance was defined a priori as *p* < 0.05.

## 3. Results

### 3.1. Patients

**Figure 1:**
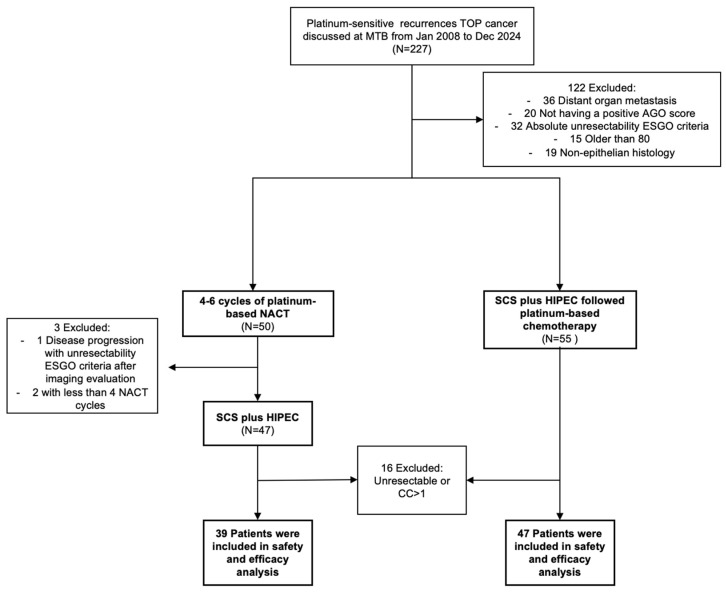
Flowchart of patient selection. Abbreviations: AGO, Arbeitsgemeinschaft Gynäkologische Onkologie; CC, Completeness Cytoreduction; ESGO, European Society Of Gynaecological Oncology; HIPEC, Hyperthermic Intraperitoneal Chemotherapy; MTB, Multidisciplinary Tumor Board; NACT, Neoadjuvant Chemotherapy; SCS, Secondary Cytoreductive Surgery; TOP, Tubal/Ovarian/Peritoneum.

Between January 2008 and December 2024, a total of 227 patients with a first platinum-sensitive recurrence of ovarian cancer were discussed at the multidisciplinary tumor board, of which 105 patients were deemed potentially resectable. Out of these, 50 patients initiated platinum-based NACT, completing 4 to 6 cycles, due to the inability to perform SCS within an optimal timeframe from diagnosis. Three patients were excluded because they did not meet resectability criteria or did not complete the four cycles of chemotherapy. Ultimately, 39 patients (45.3%) received NACT followed by SCS with hyperthermic intraperitoneal chemotherapy (HIPEC), while 47 patients (54.7%) underwent SCS with HIPEC without prior NACT. All patients had confirmation of recurrence through both frozen section and final histology. Figure 1 illustrates the study design. Baseline characteristics were comparable between groups, except for the platinum-free interval; patients with an interval exceeding 12 months predominantly belonged to the group that did not receive NACT prior to surgery (21 (53.8%) vs. 40 (85.1%), *p* = 0.001). No statistically significant differences were observed in the pattern of distribution of secondary recurrence (Table 1).

### 3.2. Survival Outcomes and Efficacy by Treatment Group

**Figure 2:**
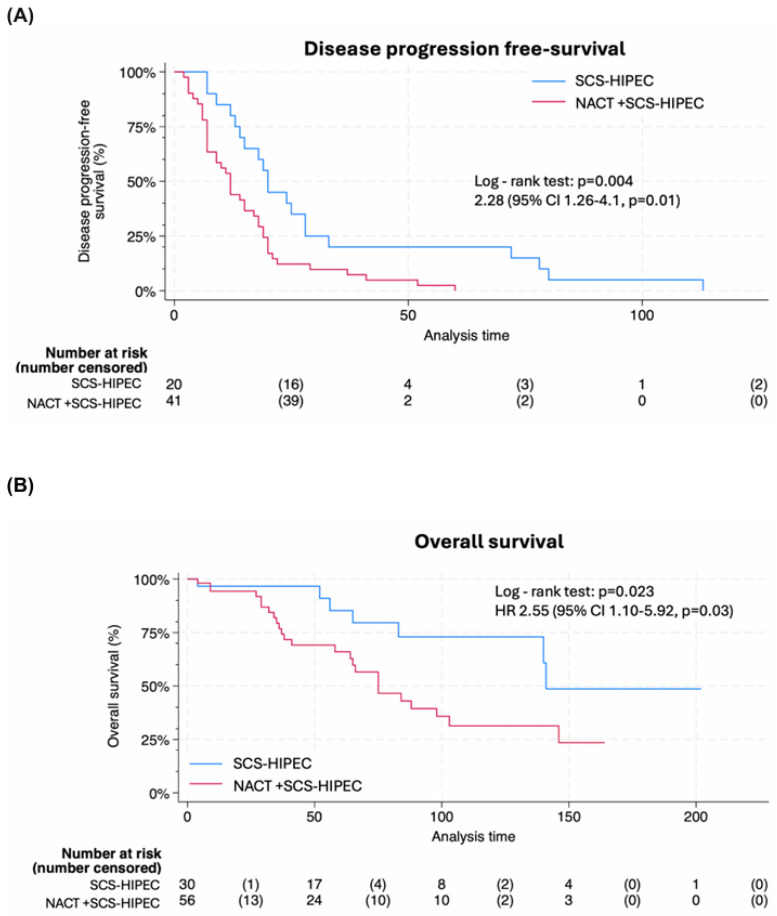

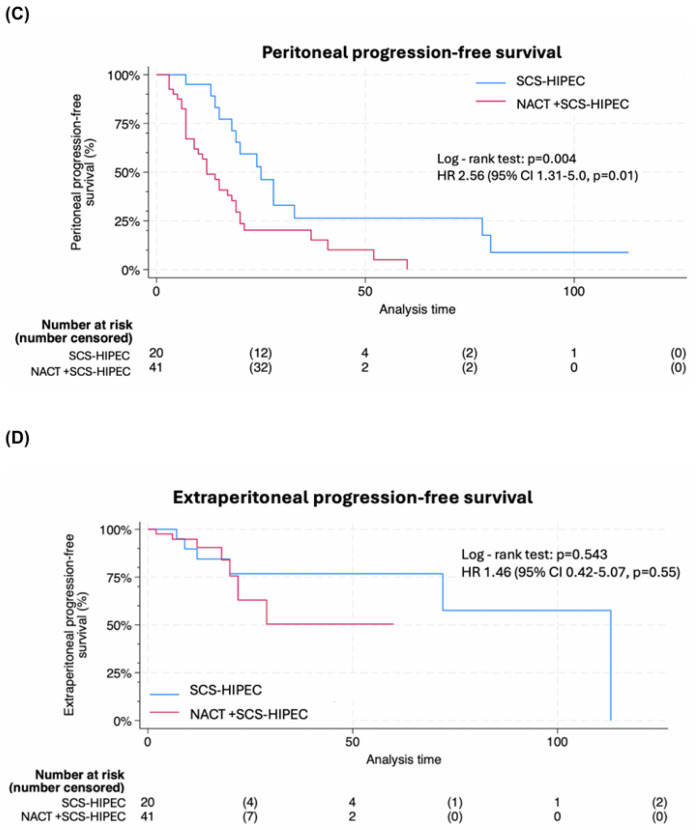
Efficacy by treatment group. (**A**) Progression-free survival. (**B**) Overall survival (**C**) Peritoneal progression-free survival. (**D**) Extraperitoneal progression-free survival. Abbreviations: HIPEC, Hyperthermic Intraperitoneal Chemotherapy; HR, Hazard Ratio; NACT, Neoadjuvant Chemotherapy; SCS, Secondary Cytoreductive Surgery.

At a median follow-up of 115 months (IQR, 54–167), patients who did not receive NACT demonstrated a significantly PFS of 20 months (95% CI, 13 to 28) compared to 12 months (95% CI, 7 to 17) for those who did receive NACT (*p* = 0.004). The hazard ratio (HR) for PFS was 2.28 (95% CI, 1.26–4.1, *p* = 0.01) (Figure 2A). Similarly, the median OS was notably better in the non-NACT group, at 88 months (95% CI, 69 to 106) versus 34 months (95% CI, 21 to 47) in the NACT group (*p* = 0.023) (Figure 2B). The HR for OS was 2.55 (95% CI, 1.10–5.92, *p* = 0.03). Additionally, analyses of peritoneal progression-free survival favored the non-NACT group; however, no significant differences were observed in extraperitoneal progression-free survival (Figure 2C,D).

**Figure 3:**
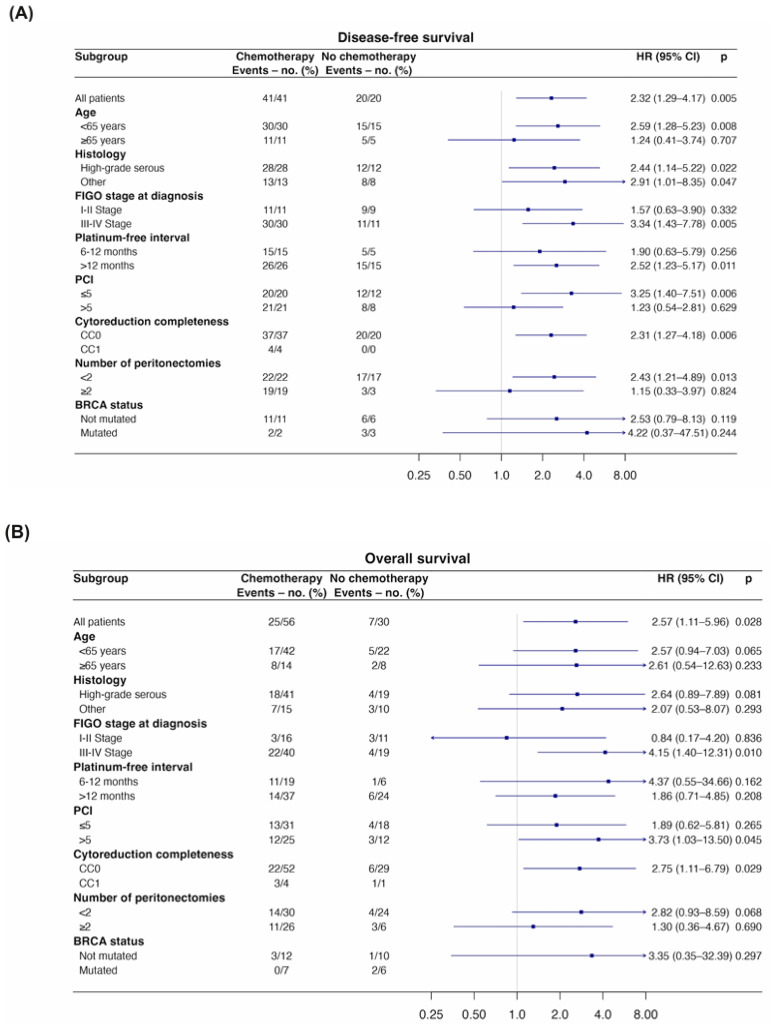

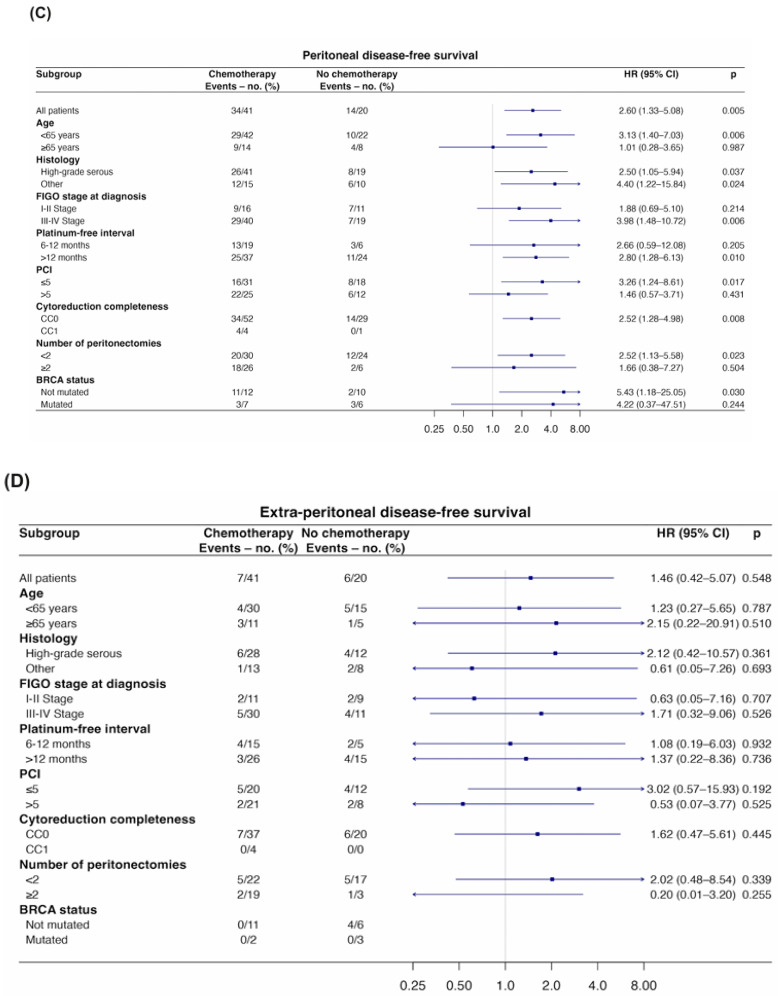
Subgroup analyses of efficacy. Forest plot for: (**A**) Progression-free survival. (**B**) Overall survival (**C**) Peritoneal progression-free survival. (**D**) Extraperitoneal progression-free survival. HR and their 95% CI are reported for each subgroup. Interaction *p*-values (unadjusted for multiple testing) are shown and should be interpreted as exploratory. Abbreviations: CI, confidence interval; HR, Hazard Ratio; PCI, Peritoneal Cancer Index.

Exploratory subgroup analyses suggested a generally consistent direction of the treatment effect across the evaluated subgroups. Although some interaction reached nominal statistical significance, these findings should be interpreted with caution given the exploratory nature of the analyses and the lack of correction for multiple testing (Figure 3). Interaction *p*-values showed numerically larger effect estimates between the non-NACT group and several subgroups, including age under 65 years, high-grade serous histology, FIGO stages III/IV, a platinum-free interval greater than 12 months, a Peritoneal Cancer Index of 5 or lower, a completeness of cytoreduction score of 0 (CC-0), and fewer than 2 peritonectomies for DFS and peritoneal disease-free survival. Additionally, the BRCA non-mutated subgroup was significant for the latter endpoint (Figure 3A,C). Notably, only the CC-0 showed a similar trend with the non-NACT group regarding overall survival, while the other subgroups exhibited a numerical trend without reaching statistical significance (Figure 3B). All these subgroups maintained a similar trend in the analysis of efficacy regarding extraperitoneal recurrences; however, the results were more heterogeneous (Figure 3D). The multivariate Cox regression analysis identified high-grade serous histology, III–IV FIGO stage at diagnosis, PCI ≤ 5 and CC-0 surgeries as independent factors that affected the DFS of patients (Table S1). Among these factors, only the FIGO stage and CC-0 surgeries demonstrated a significant impact on OS (Table S2).

### 3.3. Safety

Surgical details are presented in Table 2. In the NACT + SCS-HIPEC group, the median number of peritonectomies was significantly higher at 3 (IQR 4) compared to 1 (IQR 3) in the SCS-HIPEC group (*p* = 0.019). The median operation time was also longer in the NACT group, at 280 min (IQR 150), compared to 270 min (IQR 120) in the non-NACT group (*p* < 0.001). The incidence of blood transfusions was notably higher in the NACT group, with 15 patients (38.5%) requiring transfusions compared to just 2 patients (4.3%) in the non-NACT group (*p* < 0.001).

In the analysis of grade 3–4 complications according to the MSKCC classification, 10 patients (25.6%) were reported in the NACT group, compared to 4 patients (8.5%) in the non-NACT group (*p* = 0.040). No significant differences were observed in the rates of readmission or re-intervention (Table 3). Furthermore, a markedly higher incidence of severe complications was noted among patients receiving NACT prior to surgery, as assessed by CTCAE criteria (9 patients (23.1%) vs. 2 patients (4.3%), *p* = 0.003) (Table 4).

## 4. Discussion

### 4.1. Main Results

In this retrospective, single-center study examining the addition of 4–6 cycles of platinum-based chemotherapy to complete or optimal (residual tumor < 0.25 cm) SCS with HIPEC in women with resectable, platinum-sensitive recurrent ovarian cancer, we found that patients in the non-NACT group exhibited longer progression-free survival, overall survival, and peritoneal progression-free survival compared to those undergoing primary SCS with HIPEC alone. Secondary analyses indicated that the NACT cohort experienced more extensive surgeries, longer operative times, increased transfusion requirements, and a higher incidence of grade 3–4 complications.

### 4.2. Results in the Context of Published Literature

The DESKTOP III and SOC-1 trials demonstrated that secondary cytoreductive surgery (SCS) followed by chemotherapy is superior to chemotherapy alone for patients with recurrent epithelial ovarian cancer [8,9]. These phase III randomized studies evaluated SCS before adjuvant chemotherapy and reported findings as recruitment concluded for two [12,13] of the three [18] trials assessing the role of hyperthermic intraperitoneal chemotherapy (HIPEC) in recurrent disease contexts. The CHIPOR study [12], an international, multicenter, phase III randomized trial, provided evidence that the addition of HIPEC to SCS significantly improves overall survival (OS) in platinum-sensitive recurrent epithelial ovarian cancer patients following neoadjuvant chemotherapy (NACT). Although the median disease-free survival difference was marginal at approximately 0.7 months, OS analysis revealed a statistically significant improvement in the HIPEC group (54.3 months, 95% CI = 41.9–61.7 vs. 45.8 months, 95% CI = 38.9–54.2). The study’s design, including SCS after NACT, complicates direct prognostic comparisons with other studies. Furthermore, the lack of standardized preoperative clinical evaluations for resectability, unlike the DESKTOP III and SOC-1 trials, and the absence of essential baseline data such as initial tumor burden (Peritoneal Cancer Index) and FIGO stage at diagnosis are notable limitations. Despite these caveats, the CHIPOR trial results remain foundational and support a shift in clinical practice regarding the use of HIPEC in SCS. The MITO-18 (HORSE) [13] study was a multicenter, phase III randomized clinical trial assessing the efficacy of HIPEC in platinum-sensitive recurrent ovarian cancer patients, this time without NACT. Following surgery and assigned interventions, standard platinum-based adjuvant chemotherapy was administered to both groups. The analysis showed a median disease-free survival of 25 months in the HIPEC group (95% CI: 18–32) compared to 23 months in the control group, a difference that was not statistically significant. These findings are consistent with the earlier study by Zivanovic et al. [18]. Both studies also evaluated safety outcomes and adverse event profiles, revealing no significant differences between groups. Due to methodological variations and substantial differences in study designs, direct comparisons between CHIPOR and other studies are not feasible. It can be concluded that while HIPEC demonstrates benefits in patients undergoing SCS after NACT, it does not confer advantages in scenarios involving direct SCS. Our study is the first to compare arms from both studies, aiming to clarify the benefits of NACT induction in recurrent ovarian cancer patients opting for SCS with HIPEC. Our results demonstrate a PFS for the NACT group similar to that of the CHIPOR study arm (12 months vs. 10.2 months), as well as for the non-NACT group compared to the MITO-18 (HORSE) study arm (20 months vs. 25 months). On the other hand, it is important to clarify that this comparison is relative in the case of the treatment arm similar to the CHIPOR population, where DFS was calculated from the time of surgery following NACT, whereas in our study, it was defined from the time of recurrence diagnosis.

Complete cytoreduction during primary surgery and tumor sensitivity to platinum-based chemotherapy are key prognostic factors. HIPEC offers direct exposure of chemotherapeutic agents to the tumor, improving survival and allowing treatment across the peritoneum before adhesions form [19]. The addition of hyperthermia enhances the tumor’s response, resembling a BRCAness-like phenotype [20]. These findings support the hypothesis that HIPEC could offer clinical benefits in cases with recent chemotherapy exposure and resectable tumors, a theory corroborated in primary advanced ovarian cancer scenarios where HIPEC benefits have been observed in interval cytoreductive surgery (ICS) [15,21,22]. Koole et al. [23] showed that patients with BRCA1/2 m tumors are particularly sensitive to platinum (neoadjuvant), and HIPEC may not further enhance its effects compared to intravenous chemotherapy. This observation could be reinforced by the neoadjuvant administration of chemotherapy, which may potentially induce platinum resistance. This subgroup was particularly small (n = 34) and the number of events was low, hence more data is needed to study the effect of intraperitoneal chemotherapy and HIPEC in this specific subgroup. The unfavorable results observed in our study for the NACT group prior to SCS may be influenced for BRCA status. It is indeed the case that the BRCAm patients subgroup was minor in NACT group, which may account for observable differences in oncological outcomes. Koole et al. suggested that BRCA status and homologous recombination deficiency (HRD) should serve as critical tools in the decision-making process, not only concerning the application of HIPEC, but also in anticipation of more reliable results when determining the use of platinum-based NACT [23].

ICS in primary advanced ovarian cancer scenarios is defined as occurring after 3 to 4 cycles of platinum-based chemotherapy, followed by at least 2 to 3 additional cycles of adjuvant treatment. However, the number of chemotherapy cycles prior to ICS as defined in these trials is currently under investigation. The CHRONO trial [24], with results expected by 2028, aims to demonstrate that in patients with ovarian cancer not suitable for primary surgical cytoreduction, surgery after six cycles of neoadjuvant chemotherapy will yield better disease-free survival than cytoreductive surgery after only three cycles. In the context of our study, the scenario pertains to recurrence, where there is still insufficient evidence to define the treatment for NACT. The only study in this scenario utilizing NACT is CHIPOR, which is why we strive to ensure that our study population closely resembles it. Therefore, future studies are necessary to define NACT in the recurrence of ovarian cancer.

Our previously published data indicate that while the surgeon’s visual assessment exhibits high sensitivity and specificity, in 36% of patients analyzed during ICS with HIPEC, the histopathologically verified PCI was higher than the surgeon’s estimate. The use of prior systemic chemotherapy showed an almost significant correlation as an independent variable in this context (HR 1.93, CI 0.94–3.97, *p* = 0.074). It has been noted that preoperative chemotherapy can lead to both false negatives and false positives, with estimates around 30% for each [25]. Our findings suggest that the underestimation of residual disease may explain the significantly worse outcomes observed in the NACT cohort. Additionally, these results could be influenced by the fact that all included patients had standardized resectability criteria favoring complete secondary cytoreduction at the time of recurrence diagnosis.

Ultimately, while all patients included in our study were platinum-sensitive (with a platinum-free interval exceeding 6 months), the subgroup analysis revealed a significant benefit in DFS among patients with a platinum-free interval exceeding 12 months. Although it did not remain as an independent prognostic factor, the non-NACT included a higher percentage of patients with a platinum-free interval greater than 12 months (21 patients, 53.8% vs. 40 patients, 85.1%). This may be associated with poorer oncological outcomes. Our study utilized the AGO criteria, however, the alternative validation score, iMODEL [26], establishes a platinum-free interval of 16 months or more for favorable scoring. The CHIPOR and MITO-18 trials enrolled a highly chemosensitive population, with approximately half of the patients having a platinum-free interval of more than 18 months, and a median of over 17 months for both treatment groups, respectively. This reflection, along with our results, may lead us to question whether the scores currently used for SCS decision-making could still be applicable in patients undergoing HIPEC. Moreover, this lower platinum sensitivity in the non-NACT group could have influenced in more complex surgeries characterized by a greater number of peritonectomies, longer operative times, increased transfusion requirements, and postoperative complications. Nonetheless, the safety outcomes are similar to those reported by DESKTOP III and SOC-1 during SCS without HIPEC [8,9]. This argument supports the notion that proper patient selection for SCS is a cornerstone in managing recurrent disease and that HIPEC does not increase postoperative morbidity.

## 5. Strengths and Weaknesses

To date, this is the only study that builds on the benefits of HIPEC following the publication of CHIPOR in the context of recurrent ovarian cancer, conducting a careful comparison of selected patient groups that has not been previously performed in these trials. These strict inclusion and exclusion criteria provide additional data not reflected in the patients from the aforementioned trials, such as the FIGO stage at diagnosis, standardized resectability scoring (AGO score), and the PCI.

However, this study has several limitations. First, its retrospective, single-center design may introduce selection bias and limit the generalizability of the findings. It is important to consider that the results obtained in this study were based on conclusions drawn from the experience of a single-center with a high-volume of advanced ovarian cancer surgeries. Abel et al. [27] recently demonstrated that the combination of treatment in high-volume centers, and high utilization of NACT, was associated with the lowest 90-day surgical mortality and the longest 60-month survival for patients with advanced stage ovarian cancer.

Additionally, the study design capitalizes on a circumstantial situation where the decision to initiate NACT in this patient group was not based on standardized criteria. As a consequence, additional considerations arise, including the imbalances within the studied population. Regarding the PCI, this index in the NACT group was assessed SCS following chemotherapy treatment. An extent of disease with a PCI of 5 or lower influenced DFS and peritoneal DFS, serving as an independent prognostic factor for DFS. While the differences observed were not statistically significant between groups, the subgroup with a PCI greater than 5 was notably predominant in NACT group. A plausible explanation for our findings may be that this population harbored more extensive disease at baseline.

Finally, a significant limitation that hampers the interpretation of the results is the lengthy duration of the study period, particularly following the emergence of poly (ADP-ribose) polymerase inhibitors (PARPi). The lack of this information in our population limits the interpretation of the results. However, when comparing them with current evidence, among similar studies analyzing the surgical scenarios in recurrence, the population included in the CHIPOR study derived the most benefit from these therapies, albeit still being a minority at 20.5%, and it was residual in SOC 1, DESKTOP III, and MITO-18 (HORSE), with 10%, <5%, and <5%, respectively [8,9,12,13]. A recent retrospective study suggested that SCS followed by platinum-based chemotherapy and olaparib as maintenance therapy resulted in longer OS and DFS than SCS followed by platinum-based chemotherapy only in patients with recurrent epithelial ovarian cancer, especially in patients treated with SCS plus HIPEC [28]. It appears evident that the consideration of BRCA/HRD status and PARPi therapy will play a crucial role in the design of all future studies aimed at addressing questions related to surgery for recurrent disease.

## 6. Implications for Practice and Future Research

This study addresses an important issue that has not been previously examined regarding the role of neoadjuvant therapy in the context of surgery after recurrence of ovarian cancer. The results obtained should not be interpreted as definitive in clinical practice due to the limitations already outlined; however, they present various hypotheses to consider in the design of future studies on surgery in ovarian cancer recurrence.

Current study designs have yet to provide answers regarding the role of HIPEC in the various potential and complex scenarios of recurrent ovarian cancer. As previously mentioned, one of the advantages of hyperthermia is the sensitivity it generates for potential subsequent treatments. Conversely, a frequently criticized unresolved limitation of HIPEC is that its benefit relies on a single administration. Implementing a cyclic intraperitoneal treatment regimen would require the placement of an abdominal catheter for multiple cycles of Normothermic Intraperitoneal Chemotherapy Long Term (NIPEC-LT) post-discharge. To date, no prior study in the literature has combined NIPEC-LT following HIPEC. However, the combination of these two intraperitoneal treatment modalities appears rational, as their complications do not overlap temporally, and both have independently demonstrated survival benefits. The BICOV-1 clinical trial, a prospective, non-randomized phase I study, will evaluate the combination of HIPEC and NIPEC-LT following ICS [29].

## 7. Conclusions

In this retrospective study of patients with platinum-sensitive recurrent ovarian cancer, the addition of 4–6 cycles of platinum-based chemotherapy to secondary cytoreductive surgery (SCS) with hyperthermic intraperitoneal chemotherapy (HIPEC) did not demonstrate a statistically significant improvement in outcomes. Notably, patients who did not receive neoadjuvant chemotherapy (NACT) exhibited a trend towards better progression-free survival and overall survival, while the NACT group experienced longer surgeries and a higher incidence of complications.

Given the exploratory nature of these findings and the study design, caution is warranted in interpreting these results. While the data suggest potential differences in outcomes between the treatment groups, further research is needed to confirm these observations and to explore the implications of NACT on prognosis and surgical morbidity.

## Figures and Tables

**Table 1:** Demographic and Baseline Disease Characteristics.

Patients Characteristics	NACT+SCS-HIPEC (n = 39)	SCS-HIPEC (n = 47)	*p* Value
Age, years, median [IQR]	55 [20]	54 [20]	0.427
ASA score, n (%)			0.753
I	1 (2.6)	1 (2.1)	
II	24 (61.5)	26 (55.3)	
III	14 (35.9)	20 (42.6)	
FIGO stage, n (%)			0.909
I–II	12 (30.8)	15 (31.9)	
III–IV	39 (69.2)	32 (68.1)	
Histological type, n (%)			0.885
High-grade serous	30 (76.9)	33 (70)	
Low-grade serous	5 (12.8)	6 (12.8)	
Endometrioid	4 (10.2)	7 (14.9)	
Mucinous	0 (0)	1 (2.1)	
BRCA mutation status, n (%)			0.568
Positive	12 (30.8)	14 (29.8)	
Negative	16 (41)	28 (59.6)	
Unknown	11 (28.2)	5 (10.6)	
Second line chemotherapy, cycles completed			0.832
4–5	4 (10.2)	5 (10.7)	
6	35 (89.7)	42 (89.4)	
Platinum-free interval, months, n (%)			0.001
6–12	18 (46.2)	7 (14.9)	
>12	21 (53.8)	40 (85.1)	
Second-line chemotherapy cycles completed, median [IQR]	6 [1]	NA	
Type of recurrence, n (%)			0.158
Peritoneal only	21 (53.8)	19 (40.4)	
Extraperitoneal or mixed	18 (45.9)	28 (59.6)	
PCI, n (%)			0.159
≤5	19 (48.7)	30 (63.8)	
>5	20 (51.3)	17 (36.2)	

Abbreviations: ASA, American Society of Anesthesiologists; FIGO, International Federation of Gynecology and Obstetrics; HIPEC, hyperthermic intraperitoneal chemotherapy; IQR, Interquartile range; NA, not applicable; NACT, Neoadjuvant Chemotherapy; PCI, peritoneal cancer index; SCS, Secondary Cytoreductive Surgery.

**Table 2:** Surgical Treatment Characteristics.

Surgical Procedures	NACT+SCS-HIPEC (n = 39)	SCS-HIPEC (n = 47)	*p* Value
HIPEC regimen			0.790
Paclitaxel (60 mg/m^2^)	3 (7.7)	5 (10.6)	
Cisplatin (75 mg/m^2^)	36 (92.3)	42 (84.4)	
Number of peritonectomies [14], median [IQR]	3 [4]	1 [3]	0.019
RT at SCS, n (%)			0.498
CC-0	36 (92.3)	45 (95.7)	
CC-1	3 (7.7)	2 (4.3)	
Anastomosis, n (%)			0.106
0	20 (51.3)	31 (67.4)	
1	14 (35.9)	14 (30.4)	
2	5 (12.8)	1 (2.2)	
Operation time, minutes, median [IQR]	280 [150]	270 [120]	0.001
Number of patients who received transfusion of RBCs, n (%)	15 (38.5)	2 (4.3)	<0.001
Number of transfused RBCs per patient, median [IQR]	0 [2]	0 [0]	<0.001
Length of hospital stay, d, median [IQR]	6 [6]	6 [2]	0.383
Length of ICU stay, d, median [IQR]	1 [0]	1 [0]	0.079
Time to oral tolerance, d, median [IQR]	2 [1]	2 [1]	0.557

Abbreviations: HIPEC, Hyperthermic Intraperitoneal Chemotherapy; ICU, Intensive Care Unit; NACT, Neoadjuvant Chemotherapy; RBC, Red Blood Cell; IQR, Interquartile Range; RT, Residual Tumor; SCS, Secondary Cytoreductive Surgery.

**Table 3:** Adverse events occurring within 60 days of surgery.

	NACT+SCS-HIPEC (n = 39)	SCS-HIPEC (n = 47)	*p* Value
MSKCC grade 3–4 complications, n (%)	10 (25.6)	4 (8.5)	0.040
Events *			
Pleural effusion	1 (2.6)	0 (0)	
Bowel perforation/anastomotic leakage	3 (7.7)	2 (4.3)	
Post-operative bleeding	1 (2.6)	1 (2.1)	
Wound/abdominal wall complications	2 (5.1)	0 (0)	
Lymphocele	1 (2.6)	0 (0)	
Intrabdominal fluid collection	2 (5.1)	1 (2.1)	
Unplanned re-admission within 30 d of surgery, n (%)	4 (10.3)	5 (10.6)	0.622
Re-operation due to complications, n (%)	3 (7.7)	2 (4.3)	0.498

Abbreviations: HIPEC, hyperthermic intraperitoneal; MSKCC, Memorial Sloan Kettering Cancer Center; NACT, Neoadjuvant Chemotherapy; SCS, Secondary Cytoreductive Surgery. * Several events can occur in the same patient.

**Table 4:** Adverse events related with chemotherapy.

	Events *	NACT+SCS-HIPEC (N = 39)	SCS-HIPEC (N = 47)	*p* Value
	CTCAE grade 3–4 complications, n (%)	9 (23.1)	2 (4.3)	0.003
	Events *			
	Leukopenia	2 (5.1)	1 (2.1)	
	Trombosis	2 (5.1)	1 (2.1)	
	Thrompocytopenia	1 (2.1)	0 (0)	
	Hypoalbuminemia	2 (5.1)	0 (0)	
	Transaminase increased	1 (2.6)	0 (0)	
Pleural effusion		1 (2.6)	0 (0)	

Abbreviations: CTCAE, Common Terminology Criteria for Adverse Events; HIPEC, Hyperthermic Intraperitoneal Chemotherapy; NACT, Neoadjuvant Chemotherapy; SCS, Secondary Cytoreductive Surgery. * Several events can occur in the same patient.

## Data Availability

The original contributions presented in this study are included in the article/Supplementary Material. Further inquiries can be directed to the corresponding authors.

## Supplementary Materials

The following supporting information can be downloaded at: https://www.mdpi.com/article/10.3390/cancers18050744/s1, Table S1: Multivariate analysis of disease-free-survival according to prognostic baseline factors; Table S2: Multivariate analysis of overall survival according to prognostic baseline factors; Table S3: Treatment periods of patients included in the analysis.
