# Supplementary material for: Neoadjuvant Chemotherapy Following Hyperthermic Intraperitoneal Chemotherapy in Platinum-Sensitive Recurrent Ovarian Cancer: A Retrospective Cohort Study
Source: Cancers (Basel). 2026 Feb 26;18(5):744. doi: 10.3390/cancers18050744 (PMC12984952; doi:10.3390/cancers18050744)
Supplement: Supplementary file 1 [file cancers-18-00744-s001.zip › cancers-4147057-supplementary.pdf]

cancers

MDPI

Supplementary materials

Table S1. Multivariate analysis of disease-free-survival according to prognostic baseline factors.

	HR	IC 95%	P value
**Histology**			
High grade serous	2..07	1.3-4.8	0.012
**FIGO stage at diagnosis**			
III-IV stage	2.87	1.5-4.3	0.002
**Platinum-free interval**			
>12 months	0.58	0.32-1.04	0.07
**PCI**			
≤ 5	2.76	1.3-5.5	0.04
**Cytoreductions completeness**			
CC-0	2.04	1.9-3.7	0.001
**Number of peritonectomies**			
<2	1.98	0.6-2.9	0.14

Abbreviations: HR, Hazard Ratio; PCI, Peritoneal Cancer Index.

Table S2. Multivariate analysis of overall survival according to prognostic baseline factors.

	HR	IC 95%	P value
**FIGO stage at diagnosis**			
III-IV stage	3.7	1.9-6.9	0.01
**Platinum-free interval**			
>12 months	0.4	0.19-0.85	0.2
**PCI**			
≤ 5	2.8	0.8-7.5	0.08
**Cytoreductions completeness**			
CC-0	1.8	1.1-4.7	0.04

Abbreviations: HR, Hazard Ratio; PCI, Peritoneal Cancer Index.

Table S3. Treatment periods of patients included in the analysis.

Study group	2012-2015	2016-2020	2021-2024
NACT+SCS-HIPEC (N=39) n (%)	6 (15.4)	18 (46.2)	15 (38.5)
SCS-HIPEC (N=47), n (%)	5 (10.6)	28 (59.6)	14 (29.8)

Abbreviations: HIPEC, Hyperthermic Intraperitoneal Chemotherapy; NACT, Neoadjuvant Chemotherapy; SCS, Secondary Cytoreductive Surgery.
